# Thyroglobulin as an adjunct biomarker for assessing thyroid function during pregnancy

**DOI:** 10.5339/qmj.2025.82

**Published:** 2025-09-10

**Authors:** Terry Gbaa, Simeon Adebisi, John Bolodeoku, Faeren Dogoh, Terna Gav

**Affiliations:** 1 Department of Chemical Pathology, Benue State University Teaching Hospital, Makurdi, Benue, Nigeria; 2 Lipid Clinic, Department of Cardiology, Basingstoke and North Hampshire Hospital, Basingstoke, United Kingdom *Email: terrygbaa@yahoo.co.uk

**Keywords:** Thyroglobulin, pregnancy, thyroid function test, thyroid status, thyroid-stimulating hormone, free thyroxine

## Abstract

**Background:**

Thyroglobulin has been identified as a marker for thyroid cancer monitoring. However, researchers have proposed and employed it as a biomarker to assess iodine-dependent thyroid dysfunction during pregnancy. Pregnancy is a hyperdynamic state that significantly strains the mother’s iodine stores due to the demands of the foetus. This study combined thyroglobulin and thyroid function tests to see their impact on identifying more patients who are at risk for thyroid disorders in pregnancy. The aim of the study was to determine thyroglobulin as an adjunct biomarker in thyroid function assessment in pregnancy.

**Methods:**

Participants were across five centers, and the study was conducted over a period of 9 months (June 2019–February 2020). The study comprised a cohort of 250 pregnant women who were attending their antenatal clinic visits. These participants were selected randomly using a table of random numbers. Blood samples were taken and analyzed using immunoassay techniques. The data were analyzed using Statistical Package for Social Sciences (SPSS), version 21 (IBM, Chicago, IL).

**Results:**

Thyroid-stimulating hormone (TSH) assay only identified 35 (14%) participants, whereas the combination of the TSH and Tg assays identified 50 (20%) participants. Thyroglobulin and free thyroxine measurements revealed the presence of hyperthyroidism in 15 (9.6%) and hypothyroidism in 8 (3.2%). Using both TSH and thyroglobulin, we identified 54 (21.6%) participants as having thyroid dysfunction, with a higher prevalence of 40 (16%) hypothyroid participants compared to 14 (5.6%) hyperthyroid participants.

**Conclusion:**

Thyroglobulin is valuable during pregnancy, with the ability to reflect iodine status as a sensitive marker in identifying early thyroid dysfunction.

## 1. INTRODUCTION

Thyroglobulin (Tg) is a 660 kDa dimeric glycoprotein exclusively produced by the thyroid gland and is essential for the synthesis of thyroid hormones. Research has indicated that measuring serum levels of Tg, along with thyroxine-binding globulin, is crucial for accurately assessing thyroid gland function during pregnancy.^1^ Tg is recognised as a reliable indicator of iodine levels in the body, giving a clearer picture of iodine reserves and consumption than other thyroid function tests (TFTs).^2^ Pregnancy commonly leads to changes in thyroid function, with gestation often associated with relative hypothyroxinaemia, elevated Tg levels, and thyroid enlargement in regions with slightly low iodine intake.^3^ This could be due to the demand of the body for iodine necessary for both the mother and foetus, and may be pronounced in an iodine-deficient pregnancy, leading to pregnancy-induced hypertension, abruption, and an increase in the frequency of low birth weight infants, especially in hypothyroidism, which is seen in iodine deficiency. Tg has long been a marker for monitoring thyroid cancer; it has, however, gained value as a surrogate marker for iodine deficiency and thyroid dysfunction.

Tg levels can notably rise during pregnancy but typically return to preconception levels post-delivery.^4^ Furthermore, Tg has been validated as a biomarker for evaluating iodine status in pregnant women, showing promise for application in this demographic.^5^ The monitoring of Tg levels, alongside other thyroid function markers, is vital during pregnancy to ensure optimal health outcomes for both the mother and the foetus. It is recommended that the first trimester is the most important period of screening and diagnosis. Additionally, Tg could be measured every 4 to 6 weeks as pregnancy progresses. Tg functions as a practical biomarker of iodine status and thyroid function, offering valuable insights into thyroid activity and iodine availability for hormone synthesis. According to Mulder *et al*, the concentration of maternal Tg correlated inversely with fetal IQ, showing a higher Tg level associated with higher thyroid dysfunction and decreased fetal IQ.^6^

The understanding of the correlation between Tg and thyroid function in pregnancy is crucial for effectively managing thyroid health and safeguarding the well-being of both the mother and the developing foetus. Tg, as an essential protein for thyroid synthesis, has been considered a marker for iodine deficiency in a population.^7^ The most sensitive TFTs in pregnancy are thyroid-stimulating hormone (TSH) and free thyroxine (fT_4_), having the limitations of not identifying thyroid dysfunction early enough in pregnancy, as pregnancy is a hyperdynamic state during a woman’s reproductive life. The study aims to determine Tg as an adjunct biomarker in thyroid function assessment and to accurately stratify thyroid dysfunction in pregnancy.

## 2. METHODS

### 2.1 Study Design and Setting

This was a muticentre hospital-based study and involved a descriptive cross-sectional analysis of pregnant women. The study required the examination of data gathered from the participants throughout the course of the research. The study was conducted over a period of nine months (June 2019–February 2020). The process of enrolling pregnant women in the antenatal clinic (ANC) and collecting samples for serum TFTs, urine iodine analysis, laboratory analysis, and assessment of thyroid dysfunction in pregnancy was carried out over the specified nine months. The study sample was selected from Makurdi, a city located in North-central Nigeria.

### 2.2 Cohort

The study comprised a cohort of 250 pregnant women who were attending their ANC visits. The selection of these individuals was conducted through a random sampling process using a table of random numbers. The participants were presented with information regarding the study, obtained their signed consent, and filled out questionnaires. The participants were classified into three trimesters according to their thyroid function.

### 2.3 Inclusion Criteria

- Pregnant women in the first, second, and third trimesters attending routine ANC.
- No history of thyroid dysfunction.

### 2.4 Exclusion Criteria

- Participants who had thyroid disease or were seriously or chronically,
- Participants taking specific medications such as lithium, amiodarone, anti-seizure drugs, interferon alpha, hormone replacement therapy, that is, estrogen, and rifampicin.

### 2.5 Sample Collection and Analysis

Non-fasting samples were obtained by collecting venous blood. A volume of 5 mL of blood was drawn using a syringe and needle in a sterile manner and placed into a simple vacutainer tube. The samples were centrifuged using a tabletop centrifuge (StatSpin Express) at a speed of 3,000 revolutions per minute for a duration of 10 minutes. The resulting serum samples were then transferred into cryovials and stored at a temperature of −20°C. The serum samples were analyzed using the ultrasensitive enzyme-linked immunosorbent assay (ELISA) technique provided by Monobind Inc^®^ (AccuBind^®^ ELISA Kits, California) for the purpose of assessing thyroid function. The analysis was conducted using an automated system equipped with a microstrip reader (STAT-FAX 303).

### 2.6 Thyroglobulin Assay

Samples collected for the Tg assay were stored at −20°C before analysis. Serum Tg was measured by a quantitative enzyme-linked immunoassay (ELISA) technique using a kit from the Tg AccuBind^®^ ELISA test system supplied by Monobind Inc. It had an analytical sensitivity of 0.4 ng/mL.

### 2.7 Thyroid Function Assay

Samples collected for the thyroid function assay were stored at −20°C before analysis. A quantitative enzyme-linked immunoassay (ELISA) method was used to measure TSH/fT_4_ levels in serum. A kit from Monobind Inc’s TSH/fT_4_ AccuBind^®^ ELISA test system was used. TSH had an analytical sensitivity of 0 mIU/mL. fT_4_ demonstrated an analytical sensitivity of 0.04 ng/dL.

### 2.8 Statistical Analysis

Statistical Package for Social Sciences (SPSS), version 21 ( IBM, Chicago, IL, USA). The means were presented as mean ± SD, while categorical variables were presented as a number or percentage using descriptive statistics.

### 2.9 Ethical Consideration

This study adhered to ethical standards in accordance with the Declaration of Helsinki and was ethically approved as a component of a broader study by the Health Research Ethics Committee of Benue State University Teaching Hospital (registration code: BSUTH/CMAC/HREC/101/V.I/52). Before their enrolment in the study, each potential participant was required to provide informed and written consent. Strict confidentiality was upheld throughout the duration of the investigation.

## 3. RESULT

**Figure 1.**
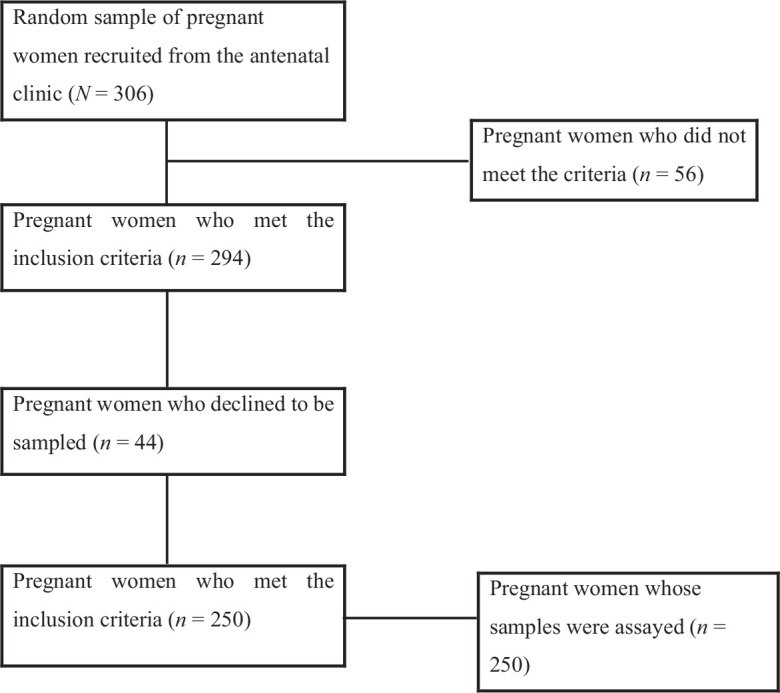
Flowchart of the recruitment of pregnant women into the study.

Using a simple random sampling technique, the study selected 306 pregnant women, 294 of whom met the study’s inclusion requirements, and 44 declined to participate in the sample. After that, we collected and analyzed blood samples from 250 participants, as seen in a flowchart in Figure 1. A total of 250 pregnant women were enrolled in the study, distributed across the three trimesters of pregnancy as follows: 51 participants in the first trimester, 114 in the second trimester, and 85 in the third trimester.

We categorised the participants into three groups based on their thyroid status: hypothyroid, euthyroid, and hyperthyroid using the TSH assay (Table 1). This distribution of thyroid dysfunction revealed a higher prevalence of hypothyroidism compared to hyperthyroidism among the study population. Specifically, 46 participants (20.8%) were classified as hypothyroid, while 35 participants (14%) were identified as hyperthyroid. These findings suggest that subclinical or overt hypothyroidism may be more common than hyperthyroidism among pregnant women in this cohort, underscoring the importance of routine thyroid function screening during pregnancy. The mean ± SD for TSH was 2.76 ± 2.80, 1.53 ± 1.49, and 3.50 ± 3.9 (mIU/L).

We assessed the thyroid status of the pregnant women in our study by utilizing a combination of fT_4_ and Tg levels. Based on this approach, we identified 15 participants (9.6%) as having hyperthyroidism and 8 participants (3.2%) as having hypothyroidism, as detailed in Table 2. However, this diagnostic strategy demonstrated inferior performance in detecting thyroid dysfunction when compared to the use of TSH alone. The lower sensitivity and specificity of the fT_4_ and Tg combination suggest that TSH remains a more reliable single biomarker for thyroid screening during pregnancy in this population.

The mean ± SD for fT_4_ was 0.75 ± 0.26, 0.80 ± 0.30, and 0.57 ± 0.23 (pg/mL) in the first, second, and third trimesters, respectively, while the mean ± SD for Tg was 21.3 ± 20.1, 22.5 ± 17.5, and 25.54 ± 24.33 (ng/mL) in the first, second, and third trimesters, respectively.

The combined use of TSH and Tg in assessing thyroid function demonstrated a slightly higher diagnostic yield for hypothyroidism compared to hyperthyroidism. Specifically, this combination identified 55% of individuals with hypothyroidism, whereas it detected 50% of those with hyperthyroidism, as presented in Table 3. These findings suggest that the combination of TSH and Tg may be more sensitive in detecting cases of underactive thyroid function than overactive thyroid function within this cohort.

## 4. DISCUSSION

Several studies have shown that Tg is important for identifying the size and function of the thyroid gland. This implies its potential use as a functional biomarker, particularly in regions with low iodine levels.^8,9^ Venables *et al* suggested the use of Tg as a sensitive indicator for evaluating thyroid function, particularly in situations involving decreased or elevated levels of iodine.^10^ Research on thyroid autoimmunity and its effects on pregnancy outcomes has highlighted the importance of Tg, further emphasizing its role in assessing thyroid function during pregnancy. Intervention studies by Benmiloud *et al* revealed the response of Tg to iodine supplementation as an indicator for the improvement in iodine status.^10^ This evidence agreed with Eltom’s research on utilising thyroglobulin as a alternative to iodine status.^11^ Another study confirmed that cord blood Tg was higher than maternal Tg concentration at 36 weeks of gestation, which showed a strong correlation with maternal iodine status.^12^

Thyroid assessment utilising TSH assay as the only screening parameter identified 76 (32.8%) hypothyroid versus hyperthyroid (41 [18.8%] vs. 35 [14%]) pregnant women, respectively. However, there was a variety of combinations of using TSH, Tg, and fT4 so as to observe the sensitivity of screening thyroid dysfunction using Tg as a common denominator. The combination of TSH and Tg assays identified 105 (42%) pregnant women with hypothyroid versus hyperthyroid (55 [22%] vs. 50 [20%]), respectively. This combination observed a 9.2% increase in pregnant women identified as having thyroid disorder, previously classified as euthyroid using only the TSH assay. Another combination involving Tg and fT4 measurements identified 27 (9.2%) hypothyroid versus hyperthyroid (8 [3.2%] vs. 15 [6%] hyperthyroid) pregnant women, respectively. Consideration should be given to iodine levels and the capacity for thyroid hormone synthesis. A TSH assay alone may not be sufficient to directly assess thyroid dysfunction in pregnancy, especially in the first trimester, as hCG may stimulate the thyroid receptors owing to it sharing the same alpha receptor as TSH. This study observed that these assays, especially TSH and Tg, offered a more precise assessment of thyroid function. However, the combination of fT_4_ and Tg showed a lower screening yield in pregnancy.

This implies that the combination of Tg and TSH can detect minor thyroid abnormalities that may go unnoticed when using a single thyroid biomarker. The wide range of classifications highlights the drawbacks of simply depending on only TSH concentration, which can vary due to multiple variables and may not consistently indicate actual thyroid well-being. Therefore, including Tg in routine thyroid assessment could improve the diagnostic accuracy in pregnancy-associated thyroid dysfunction.

This strategy could facilitate the timely identification and treatment of thyroid dysfunction, which is crucial during pregnancy to prevent unfavorable consequences for both the mother and foetus. The combination of TSH and Tg identified 54 pregnant women (21.6%) as having thyroid dysfunction. Among these, hypothyroidism was more prevalent, with 40 (16%) affected, compared to 14 (5.6%) who were classified as hyperthyroid. In comparison, the use of the TSH assay alone identified a total of 43 (29.6%) pregnant women with thyroid dysfunction, which included 36 (16.8%) hypothyroid and 7 (6.8%) hyperthyroid. Notably, all these participants were in their second trimester of pregnancy.

This indicates that a greater number of pregnant women have a higher likelihood of developing hypothyroidism in the second trimester. This could be due to the increased iodine in the mother due to the transplacental transfer of iodine to the growing foetus. Zhang *et al* discovered that Tg levels rise during pregnancy, highlighting the importance of considering the influence of pregnancy when using Tg as a biomarker for iodine status.^13^ Stagnaro-Green’s investigation found that the occurrence of postpartum thyroiditis ranged from 11 to 16.7%.^14^ This is in keeping with the result of this study identifying more participants with hypothyroidism. Conversely, Othman *et al* discovered that 23% of pregnant women developed postpartum thyroiditis during a span of 2 to 4 years after parturition.^15^ Thyroid dysfunction during pregnancy can result in complications such as preeclampsia, premature birth, and poor neurodevelopment in the baby, especially in hypothyroidism.^16^

In iodine-deficient individuals, the thyroid gland compensates for its activity by increasing its stimulatory effects on thyroid function.^17^ Studies have shown that Tg activity and concentration are influenced by the levels of iodine in a pregnant woman.^18^ Tg is a functional marker of iodine stores that lasts much longer than urine iodine concentration.^19^ Pregnant women supplemented with iodine saw a 27% decrease in Tg levels, compared to a 17% increase in the placebo group.^20^ Additionally, the United Kingdom has proven Tg to be a promising biomarker of iodine status during pregnancy.^21^

By incorporating Tg measurements in regular TFT, a more precise evaluation of thyroid function may be ascertained, facilitating prompt interventions. This is especially important in communities with limited resources, where iodine shortages are widespread and conventional TFTs may not provide a comprehensive assessment.

## 5. CONCLUSION

This study underscores the value of Tg as an adjunct biomarker in assessing thyroid function during pregnancy. Tg’s ability to reflect iodine status and thyroid hormone synthesis provides a more nuanced understanding of thyroid health, complementing traditional markers like TSH and fT_4_, which may not be as sensitive in identifying early thyroid dysfunction.

## 6. STUDY STRENGTH

The strength of this study was the ability to identify participants who had subclinical or overt thyroid dysfunction that was not identified using TFTs only. The integration of Tg into thyroid function assessments may lead to better diagnostic accuracy, early intervention, and improved maternal and fetal health outcomes. This study was conducted to identify participants who may be at risk of iodine deficiency and may not have sufficient iodine supplied during pregnancy. Further research and broader implementation of this combined diagnostic approach are warranted to optimize thyroid health management in pregnant women, particularly in regions with marginal iodine deficiency.

## 7. LIMITATION

Tg antibodies were not assayed, which could have been important because of their nature to interfere with the Tg assay. This could falsely elevate or decrease Tg given results that may not be precise. Every Tg assay should include a panel for thyroid autoantibodies.

## LIST OF ABBREVIATIONS

**Table:** 

ANC	Antenatal clinic
BSUTH	Benue State University Teaching Hospital
ELISA	Enzyme-linked immunosorbent assay
FT_4_	Free thyroxine
hCG	Human chorionic gonadotropin
SPSS	Statistical Package for Social Sciences
TFT	Thyroid function test
TG	Thyroglobulin
TSH	Thyroid-stimulating hormone

## AVAILABILITY OF DATA AND MATERIALS

The data collected during the current study are available from the corresponding author on reasonable request.

## AUTHORS CONTRIBUTIONS

TG and SA conceptualized the study and wrote up the manuscript. JB was responsible for proofreading the manuscript. For analysis, FD and TGa collected all the results and data. All authors reviewed and agreed with the final version of the manuscript. TG was responsible for submitting the manuscript for publication.

## ACKNOWLEDGMENTS

The authors would like to acknowledge all who have contributed immensely to this study, especially to Prof. S.A. Adebisi, Dr. P.H.O. Amodu, Dr. J. Ngbea, Prof. T. Swende, Prof. O.O. Alao, and Dr. B.K. Myke-Mbata (H.O.D.), and to all residents in Chemical Pathology.

## CONFLICT OF INTEREST

The authors declare that they have no competing interests.

## Figures and Tables

**Table 1.** Thyroid status of pregnant women classified using thyroid-stimulating hormone only (*n* = 250).

Trimester	*n* (%)	Thyroid status		
		Hypothyroid	Euthyroid	Hyperthyroid
First	51 (20.4)	4 (1.6)	42 (16.8)	5 (2.0)
Second	114 (45.6)	36 (16.8).	71 (28.4)	7 (2.8)
Third	85 (34)	1 (0.4)	56 (22.4)	23 (9.2)

**Table 2.** Thyroid status of pregnant women classified using a combination of free thyroxine and thyroglobulin (*n* = 250).

Trimester	*n* (%)	Thyroid status		
		Hypothyroid	Euthyroid	Hyperthyroid
First	51 (20.4)	3 (1.2)	46 (18.4)	2 (0.8)
Second	114 (45.6)	4 (1.6)	103 (41.2)	7 (2.8)
Third	85 (34)	1 (0.4)	78 (31.2)	6 (2.4)

**Table 3.** Thyroid status of pregnant women classified using a combination of thyroid-stimulating hormone and thyroglobulin (*n* = 250).

Trimester	*n* (%)	Thyroid status		
		Hypothyroid	Euthyroid	Hyperthyroid
First	51 (20.4)	7 (2.8)	37 (14.8)	7 (2.8)
Second	114 (45.6)	40 (16)	60 (24)	14 (5.6)
Third	85 (34)	8 (3.2)	48 (19.2)	29 (11.6)
